# Cisd2 slows down liver aging and attenuates age‐related metabolic dysfunction in male mice

**DOI:** 10.1111/acel.13523

**Published:** 2021-11-22

**Authors:** Yi‐Long Huang, Zhao‐Qing Shen, Chen‐Hua Huang, Chao‐Hsiung Lin, Ting‐Fen Tsai

**Affiliations:** ^1^ Department of Life Sciences and Institute of Genome Sciences National Yang Ming Chiao Tung University Taipei Taiwan; ^2^ Aging and Health Research Center National Yang Ming Chiao Tung University Taipei Taiwan; ^3^ Institute of Molecular and Genomic Medicine National Health Research Institutes Zhunan Taiwan

**Keywords:** Cisd2, fibrosis, liver aging, non‐alcoholic fatty liver disease, oxidative stress, RNA sequencing, transcriptomics

## Abstract

The liver plays a pivotal role in mammalian aging. However, the mechanisms underlying liver aging remain unclear. Cisd2 is a pro‐longevity gene in mice. Cisd2 mediates lifespan and healthspan via regulation of calcium homeostasis and mitochondrial functioning. Intriguingly, the protein level of Cisd2 is significantly decreased by about 50% in the livers of old male mice. This down‐regulation of Cisd2 may result in the aging liver exhibiting non‐alcoholic fatty liver disease (NAFLD) phenotype. Here, we use Cisd2 transgenic mice to investigate whether maintaining Cisd2 protein at a persistently high level is able to slow down liver aging. Our study identifies four major discoveries. Firstly, that Cisd2 expression attenuates age‐related dysregulation of lipid metabolism and other pathological abnormalities. Secondly, revealed by RNA sequencing analysis, the livers of old male mice undergo extensive transcriptomic alterations, and these are associated with steatosis, hepatitis, fibrosis, and xenobiotic detoxification. Intriguingly, a youthful transcriptomic profile, like that of young 3‐month‐old mice, was found in old Cisd2 transgenic male mice at 26 months old. Thirdly, Cisd2 suppresses the age‐associated dysregulation of various transcription regulators (Nrf2, IL‐6, and Hnf4a), which keeps the transcriptional network in a normal pattern. Finally, a high level of Cisd2 protein protects the liver from oxidative stress, and this is associated with a reduction in mitochondrial DNA deletions. These findings demonstrate that Cisd2 is a promising target for the development of therapeutic agents that, by bringing about an effective enhancement of Cisd2 expression, will slow down liver aging.

## Graphical abstract

The protein level of CDGSH iron‐sulfur domain‐containing protein 2 (Cisd2) is decreased by about 50% in the aged livers of old mice. RNA sequencing and histopathological analyses reveal that in the Cisd2 transgenic mice, a persistently high level of Cisd2 slows down liver aging via attenuating oxidative stress and mitochondrial DNA deletion, as well as preserving a youthful transcriptomic profile, thereby protecting the liver against age‐associated structural injury and functional decline.

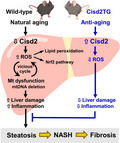

## 1 INTRODUCTION

Elderly individuals present with a higher susceptibility to acute liver injury, liver viral infections, and non‐alcoholic fatty liver disease (NAFLD), the latter being mainly manifested as an excessive accumulation of fat in hepatocytes. In addition, due to their decreased liver regenerative capacity and immune response dysfunction, elderly individuals are also more prone to liver fibrosis progression. Chronic steatosis and hepatitis have been proposed to contribute to fibrosis/cirrhosis and possible HCC development (Anstee et al., 2019).

Although the precise etiology of liver aging remains elusive, a number of studies have indicated that CDGSH iron–sulfur domain‐containing protein 2 (Cisd2) plays a pivotal role in regulating lifespan and aging‐associated organ deterioration. A deficiency in Cisd2 has been found to lead to a shortened lifespan (Chen et al., 2009a). Conversely, enhanced expression of Cisd2 promotes longevity, exerts an anti‐aging effect, and ameliorates aging‐associated phenotypes (Chen et al., 2009a, 2010; Wu et al., 2012). Furthermore, our previous study has shown that Cisd2 haploinsufficiency predisposes mice to NAFLD and HCC, especially under HBx challenge. Overexpression of Cisd2 was found to have beneficial effects and improve HBx‐mediated hepatic lipid accumulation, protect against lipotoxicity, and protect against hepatocarcinogenesis (Shen et al., 2017, 2018). Moreover, numerous studies have further confirmed that Cisd2 mitigates age‐related functional decline in a wide range of tissues, including skeletal muscle, neurons, skin, and heart (Wu et al., 2012; Yeh et al., 2019, 2020). Additionally, Cisd2 is mainly located in the ER, the mitochondrial outer membrane, and the mitochondria‐associated ER membrane (MAM). In terms of function, Cisd2 plays a pivotal role in the maintenance of Ca^2+^ homeostasis and is involved in modulating mitochondrial function and oxidative stress (Shen et al., 2021; Wang et al., 2014). Thus, Cisd2 is able to exert a wide range of cytoprotective actions via the regulation of the ER and of mitochondrial functionality; these scenarios have been postulated as the way that Cisd2 brings about extensions of lifespan and healthspan (Shen et al., 2021).

The synergistic effects of multiple factors, including insulin resistance, adipose dysregulation, inflammation, and oxidative stress, have been shown to be involved in age‐related NAFLD progression (Marjot et al., 2020; Tilg & Moschen, 2010). A number of recent studies have shown that reactive oxygen species (ROS) and mitochondrial dysfunction play a vital role in this process (Chakravarthy & Neuschwander‐Tetri, 2020; Chen et al., 2020). Although oxidative stress has been implicated in the progression of NAFLD through its various stages, it is still unclear how ROS triggers hepatic inflammation and fibrogenesis during aging. It is thought that a large amount of ROS might result in lipid peroxidation, inflammation, protein oxidation, and various types of cell death, and that these collectively contribute to the onset and development of NAFLD (Masarone et al., 2018).

The protein level of Cisd2 was found to be dramatically decreased by about 50% in the aged livers of old mice at 26 months old compared with young mice at 3 months old. This age‐dependent down‐regulation of Cisd2 was correlated with a NAFLD phenotype in the aging liver. The present study was designed to apply a genetic approach to investigate whether maintaining the Cisd2 protein at a persistently high level during old age, namely a level comparable to the Cisd2 level at a young age, is able to slow down liver aging, attenuate age‐related functional decline and mitigate structural damage; this was investigated using the livers of Cisd2 transgenic (Cisd2TG) mice that carry two additional copies of the Cisd2 gene. Furthermore, we carry out transcriptomic analysis to pinpoint the potential mechanism(s) of action associated with the anti‐aging effect of Cisd2 on the liver. Our findings provide evidence showing that providing a high level of Cisd2 (about a twofold increase) throughout a mouse's lifetime is able to preserve a youthful transcriptomic profile in the liver and slow down liver aging.

## 2 RESULTS

### 2.1 Cisd2 maintains the structure of the liver and protects the liver from age‐related pathological damage

**FIGURE 1:**
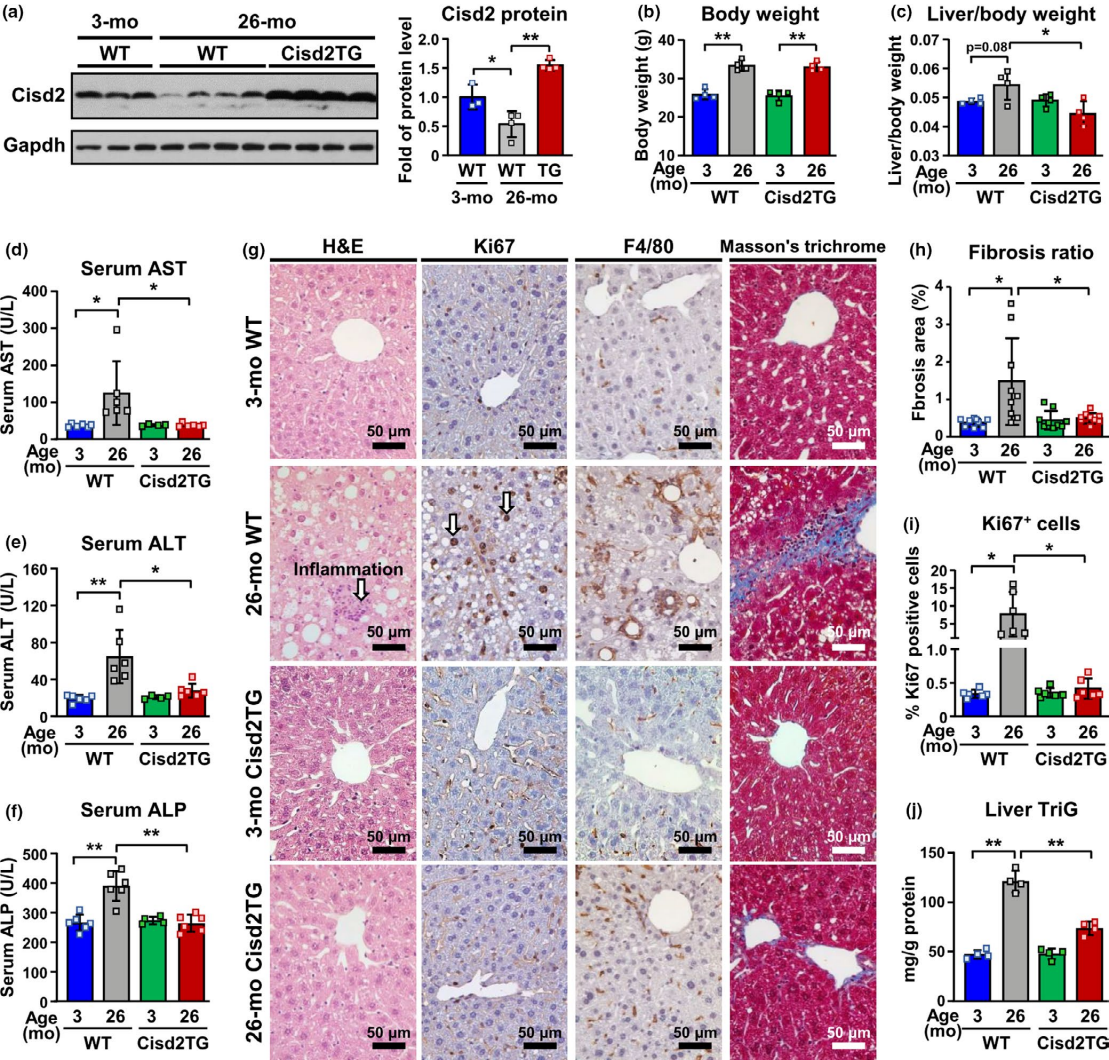
High level of Cisd2 protects the liver from age‐related pathological changes, namely hepatic steatosis, inflammation, and fibrosis during natural aging. (a) Immunoblotting of Cisd2 protein in the livers of wild‐type (WT) mice and Cisd2TG mice at 3‐mo or 26‐mo old (*n* = 3–4). (b) Body weight and (c) ratios of liver/body weight. (d) Serum AST levels (*n* = 6). (e) Serum ALT levels (*n* = 6). (f) Serum ALP levels (*n* = 6). (g) The liver sections of 3‐mo WT, 3‐mo Cisd2TG, 26‐mo WT, and 26‐mo Cisd2TG mice stained with H&E, IHC of F4/80 (Kupffer cell marker) or Ki67, and Masson's trichrome. Overt fat deposition, hepatocyte ballooning, inflammation (arrow), and fibrosis were detected in the naturally aged livers. (h) Quantitative analysis of liver fibrosis was performed by measuring the positive area of Masson's trichrome staining. For each mouse, three micrographs (100×) were used for the quantification (*n* = 3). (i) Quantitative analysis of nuclear Ki67‐positive hepatocytes. For each mouse, three micrographs (400×) were used for the quantification. (j) Total triglyceride (TriG) levels in the liver tissues. See Figure S2 for microphotographs at low magnification. Scale bar, 50 μm. Data are presented as mean ± SD. *p < 0.05; **p < 0.005. AST, aspartate aminotransferase; ALT, alanine aminotransferase; ALP, alkaline phosphatase

To study the role of Cisd2 during liver aging, the expression of the Cisd2 was assessed by immunoblotting of liver samples from old Cisd2 transgenic (Cisd2TG) mice and old wild‐type (WT) mice. At 26 months old, the WT mice were found to have undergone about a 46% reduction in Cisd2 protein in the liver compared to young WT mice at 3 months old (Figure 1a). On the contrary, a persistently high level of Cisd2 protein (about twofold) was found to be maintained in the livers of Cisd2TG mice throughout their lifetime (Figure 1a; Figure S1a). Although the body weights of the old WT and old Cisd2TG mice were similar (Figure 1b), the liver‐to‐body weight ratio was found to be significantly decreased in the old Cisd2TG mice compared to that in the old WT mice (Figure 1c), which suggests that a high level of Cisd2 suppresses hepatic fat deposition during aging. In addition, aging was found to be associated with elevated serum levels of various liver damage markers, namely aspartate aminotransferase (AST), alanine aminotransferase (ALT), and alkaline phosphatase (ALP); all of these liver damage markers were significantly lower in the old Cisd2TG mice compared to the old WT mice (Figure 1d–f). The aged livers of old mice display a combination of hepatic steatosis, macrophage infiltration, and fibrosis (Figure 1g,h). Strikingly, Cisd2 overexpression was able to suppress the development of these pathological changes (Figure 1g,h; Figure S2). Similar results were found when the percentage of proliferation marker Ki67‐positive hepatocytes was quantified. In the aged liver of old mice, there was found to be a significant increase in Ki67‐positive hepatocytes; on the contrary, hepatocyte proliferation was barely detectable in the livers of young WT, young Cisd2TG, and old Cisd2TG mice (Figure 1g,i). Furthermore, Cisd2 appears to attenuate the increase of triglyceride (TriG) associated with old livers (Figure 1j). These findings indicate that enhanced Cisd2 expression appears to delay liver aging and, furthermore, is able to ameliorate age‐related pathological damage.

### 2.2 Cisd2 retains a youthful transcriptomic profile pattern in the liver during natural aging

**FIGURE 2:**
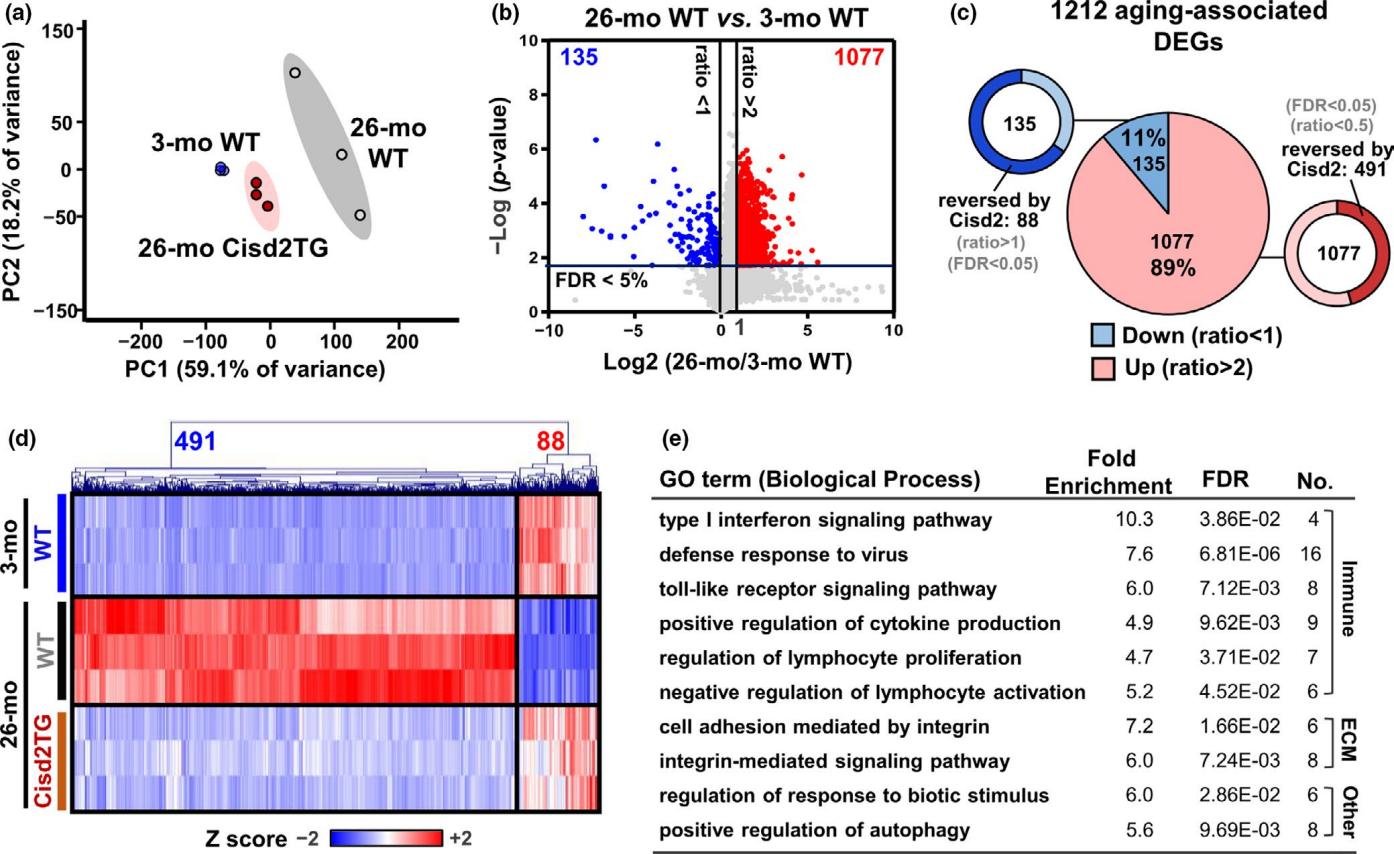
High level of Cisd2 preserves a young transcriptomic pattern in the liver of old Cisd2TG mice. (a) Principal component analysis (PCA) of RNA‐seq data for mice made up of three groups (3‐mo WT mice, 26‐mo WT mice, and 26‐mo Cisd2TG mice). (b) Volcano plot showing transcriptome change in 26‐mo vs. 3‐mo WT mice. Horizontal line shows the 5% false discovery rate (FDR) threshold. Red or blue plots display genes above the indicated FDR and fold change threshold. (c) Pie chart shows the number of mRNAs that are differentially expressed in 26‐mo WT compared to 3‐mo WT mice. Blue indicates the down‐regulated genes associated with aging in the liver, and red indicates the up‐regulated genes associated with aging in liver. The mRNAs that underwent differential reversal on overexpression of Cisd2 in the Cisd2TG mice were identified. (d) Heatmap representing the age‐dependent 579 DEGs that had their expression changes attenuated by overexpression of Cisd2 in the Cisd2TG mice. (e) Over‐represented PANTHER GO‐Slim Biological Processes of transcriptome changes during aging (1212 DEGs). The top 10 GO processes are shown

To identify the molecular basis of Cisd2‐mediated protection against liver aging, we used RNA sequencing (RNA‐seq) technology to generate the transcriptomic profiles of livers from old Cisd2TG mice at 26 months old and we compared these profiles with those of WT mice at 3 months old (young) and at 26 months old (natural aging). The expression levels of 11,316 genes were quantified in this study. Principal component analysis (PCA) showed a dramatic difference in the transcriptome profiles of young (3‐mo) WT mice and old (26‐mo) WT mice (Figure 2a). Intriguingly, the transcriptomic profile of Cisd2TG (26‐mo) mice clusters together with and is very close to that of the young (3‐mo) WT mice, suggesting that a high level of Cisd2 protein appears to delay liver aging. A total of 1,212 genes were found to be significantly up‐regulated or down‐regulated with age (Figure 2b). Of these, 491 of the 1,077 up‐regulated genes and 88 of the 135 down‐regulated genes were reversed in Cisd2TG mouse liver at 26 months (Figure 2c,d). During liver aging, six of the top ten significantly enriched Gene Ontology (GO) categories of the differential genes were associated with inflammatory processes (e.g., the type I interferon signaling pathway, defense response to virus, and the toll‐like receptor signaling pathway) (Figure 2e), which indicates that inflammation seems to be one of the critical determinants during liver aging.

### 2.3 Cisd2 counteracts the age‐associated changes that affect the liver transcriptome with respect to cellular functions, pathways, and pathology

**FIGURE 3:**
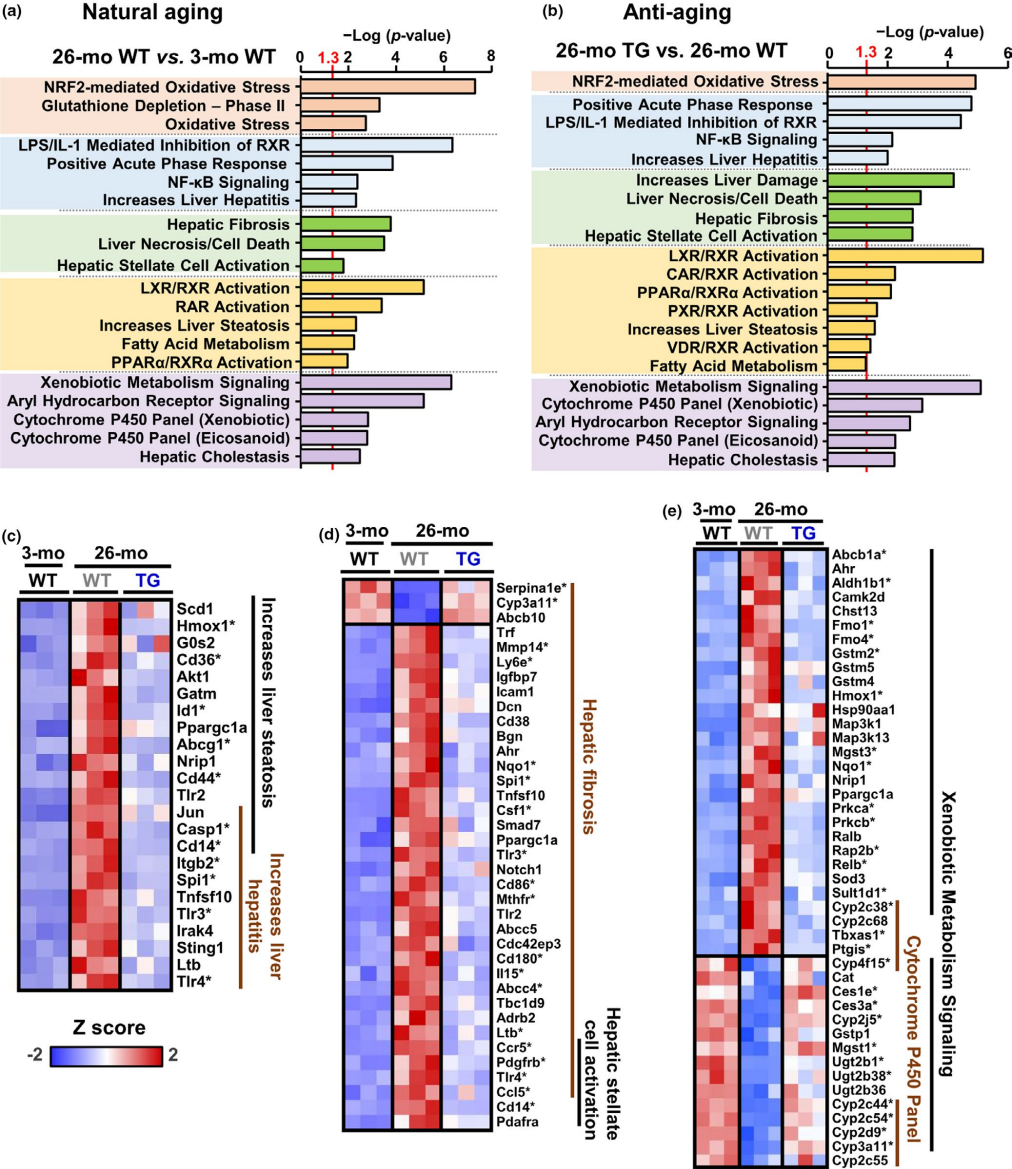
Functional analyses of the differential expression genes (DEGs), the expression patterns of which are affected by aging and the pattern of which are reversed by overexpression of Cisd2 in the Cisd2TG mice. (a, b) Ingenuity pathway analysis (IPA) toxicity list linking the RNA‐seq data in the livers of 26‐mo WT mice (a) and 26‐mo Cisd2TG mice (b) with the cellular functions, pathways, and pathological phenotypes. The highest enriched functions related to liver are grouped into various different categories, namely oxidative stress, inflammation, fibrosis, steatosis, and xenobiotic metabolism. (c–e) Heat maps depicting expression of age‐associated DEGs related to liver steatosis (c), fibrosis (d), and xenobiotic metabolism (e) (*n* = 3). The age‐related DEGs (26‐mo vs. 3‐mo) that are significantly reversed in the Cisd2TG mice are marked with an asterisk (FDR < 0.05)

Ingenuity pathway analysis (IPA) identified several significant pathways (*p* < 0.05) when the following paired comparisons were made: (a) natural aging (26‐mo WT vs. 3‐mo WT), and (b) anti‐aging (26‐mo Cisd2TG vs. 26‐mo WT). There are five major functional groupings within these pathways, and these are as follows: (1) oxidative stress; (2) inflammation/hepatitis; (3) hepatic fibrosis; (4) lipid metabolism; and (5) xenobiotic metabolism signaling (Figure 3a,b). All five of these are of interest when investigating mouse liver phenotypes. Specifically, for lipid metabolism, the levels of the genes involved in fatty acid biosynthesis (Scd1), fatty acid transport (Cd36, Abcg1), and fatty acid transcription regulation (Ppargc1a/PGC‐1α, Id1) are significantly increased upon aging. Remarkably, enhanced Cisd2 expression in the liver of Cisd2TG mice significantly lowered the expression levels of these genes, which are also known to be involved in the pathogenesis of NAFLD (Figure 3c). Additionally, in the livers of Cisd2TG mice, most of the age‐related differentially expressed genes (DEGs) that are associated with fibrosis and stellate cell activation (Figure 3d), as well as with xenobiotic metabolism and the cytochrome P450 panel (Figure 3e), still have a young pattern that is similar to that found in 3‐mo WT mice. Taken together, both PCA and the IPA data indicate that age‐related impairment of liver metabolic function can be significantly attenuated by an enhanced level of Cisd2 expression and that a youthful pattern of transcriptome is preserved in the liver of Cisd2TG mice.

### 2.4 Cisd2 attenuates aging‐induced dysregulation of various transcriptional networks

**FIGURE 4:**
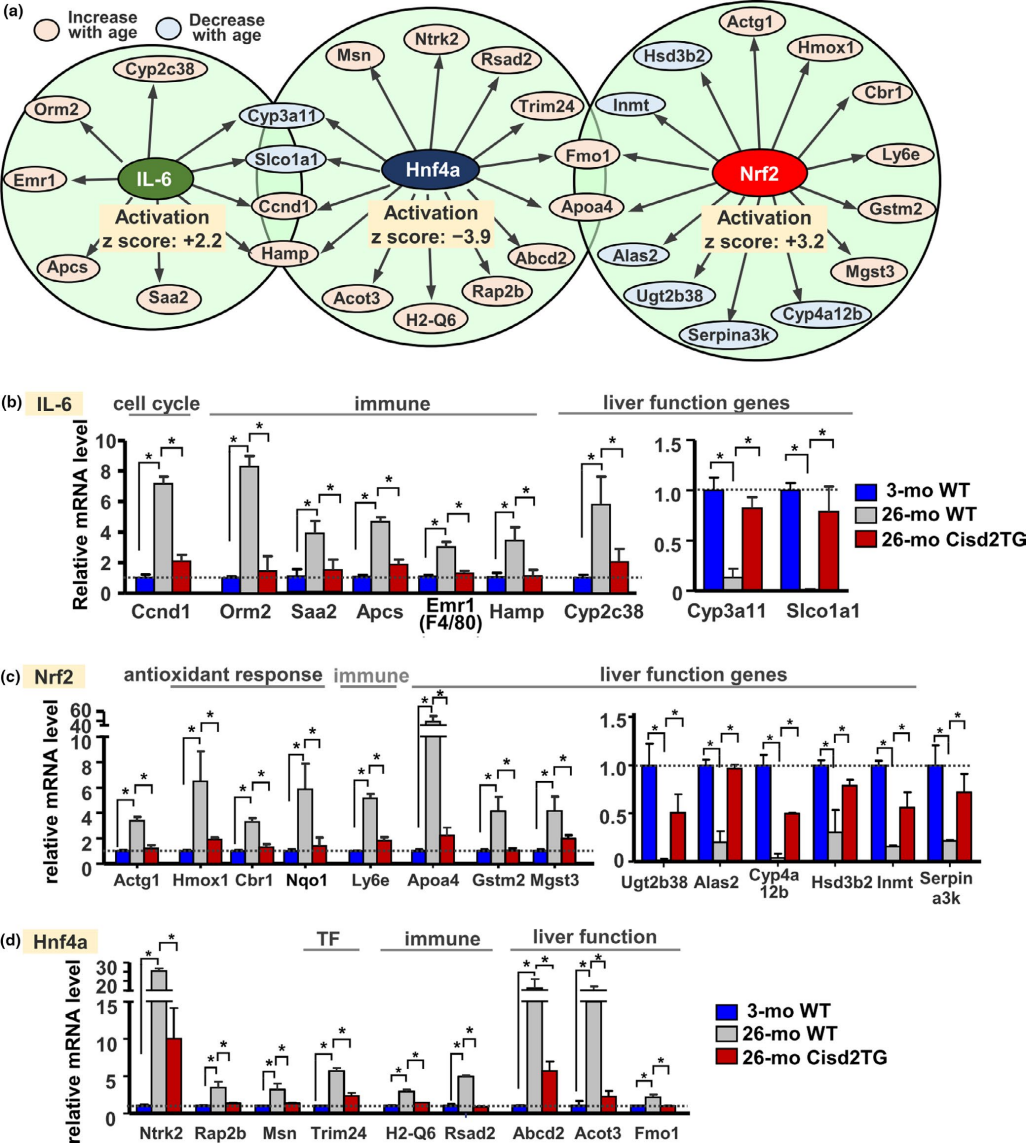
Age‐dependent dysregulation of various upstream transcription regulators and their downstream target genes is attenuated in the livers of Cisd2TG mice. (a) Significantly activated or inhibited upstream regulators based on activation *z*‐score (absolute *z*‐score >2.0) from IPA upstream regulator analysis. (b) Interleukin‐6 (IL‐6) (c) Nrf2 (d) Hnf4a‐mediated transcriptional changes in gene expression. TF, transcription factor. See Table S1 for complete gene list

Using the upstream regulator analysis routine of the IPA program, we were able to predict that Cisd2 expression at a level similar to that found in young WT mice affected a number of upstream regulators and that these regulators play important roles in attenuating liver steatosis. A total of seven down‐regulated and five up‐regulated transcription regulators were identified (*p* < 0.05 & absolute *Z* score >2; Table S1). Interleukin‐6 (IL‐6) and nuclear factor E2‐related factor 2 (Nrf2) were identified as being significantly activated (activation *z*‐score >2) upstream regulators; on the contrary, hepatocyte nuclear factor‐4α (Hnf4a) was significantly inhibited (activation *z*‐score < −2, Figure 4a). In the old WT mice (26‐mo), aging may increase IL‐6‐mediated transcriptional activity; consequently, the expression of several of its downstream target genes, such as Orm2, Saa2, Apcs, and Emr1, was increased (Figure 4b). In fact, one of the IL‐6 potential downstream target genes, Emr1 (F4/80), is the macrophage marker used in the immunostaining (Figure 1g). As shown in Figure 4c, Nrf2 appears to activate a variety of genes that are involved in the adaptive response to oxidative/xenobiotic stress. This effect could be a compensatory response to the increased level of damage present during liver aging. Notably, IPA also revealed that Hnf4a, which is a master regulator of hepatocyte differentiation and a key determinant for liver function (Lu, 2016), was significantly suppressed in the aged liver of 26‐mo WT mice (Figure 4d; Table S1). Importantly, in the liver of old Cisd2TG (26‐mo) mice, a high level of Cisd2 protein maintains these three transcription regulators, namely IL‐6, Nrf2, and Hnf4a, in a normal young pattern, thus preventing their downstream target genes from undergoing abnormal up‐regulation or down‐regulation (Figure 4b–d; Table S2). As a result, a younger liver is likely to be preserved via the maintenance of a normal transcriptional network in the old Cisd2TG mice.

### 2.5 Cisd2 suppresses age‐related oxidative stress in the liver

**FIGURE 5:**
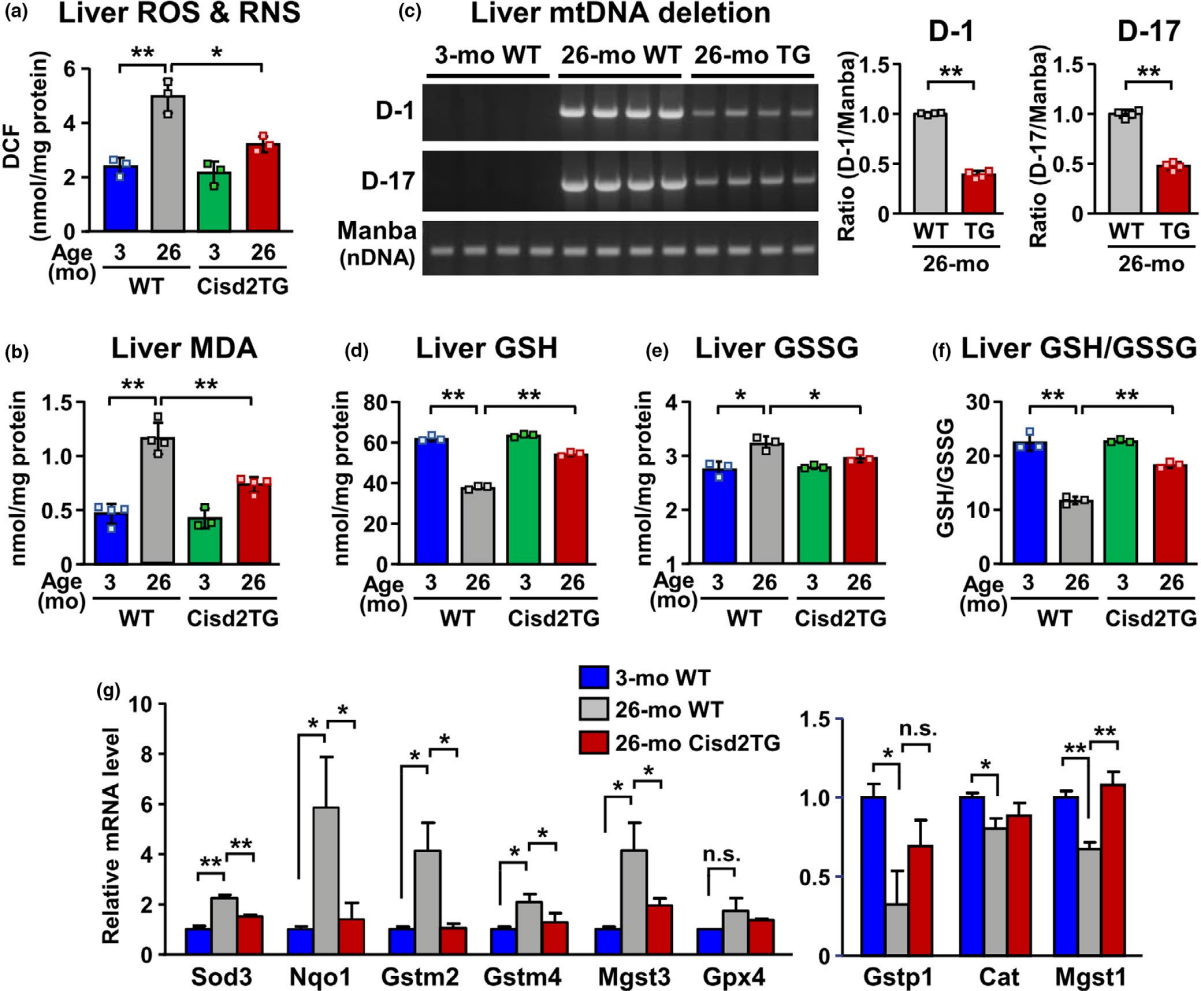
Negative correlation of Cisd2 levels with age‐related oxidative stress in the liver. (a) Total reactive oxygen and nitrogen species levels (ROS/RNS) in the liver tissues of old WT mice and old Cisd2TG mice (*n* = 3). (b) Hepatic contents of malondialdehyde (MDA). (*n* = 3–4). (c) Deletions of the mitochondrial genome (mtDNA) are attenuated in liver tissue of old Cisd2TG mice compared to old WT mice. Manba is used as the control nuclear gene (*n* = 4). (d) The reduced form of glutathione (GSH) levels in the liver tissues of old WT mice and old Cisd2TG mice (*n* = 3). (e) The oxidized form of glutathione (GSSG) levels in the liver tissues of old WT mice and old Cisd2TG mice (*n* = 3). (f) GSH/GSSG ratios in the liver tissues of WT mice and Cisd2TG mice at 3 months or 26 months old (*n* = 3). (g) Differentially expressed genes that are involved in the antioxidant defense of the livers of naturally aged mice. The young WT control group was set to have a value of 1. DCF, 2’, 7'‐dichlorodihydrofluorescein. Data are presented as mean ± SD; not significant (n.s.). *p < 0.05; **p < 0.005

To study whether an increased level of Cisd2 has a beneficial effect on the suppression of oxidative stress and liver injury, we examined ROS/RNS levels, as well as the markers of ROS‐mediated damage, in the livers of WT and Cisd2TG mice. Remarkably, there is a significant increase in the total ROS/RNS level and the level of lipid oxidation product malondialdehyde (MDA) in the aged liver of old WT mice, which indicates that the antioxidant capacity of the liver declines with age (Figure 5a,b). ROS‐mediated damage is known to be associated with liver dysfunction and may cause overt pathology in the aging liver. For example, ROS can generate double‐strand breaks and large mitochondrial DNA (mtDNA) deletions and these have been associated with mitochondrial dysfunction and aging (Chocron et al., 2019; Nissanka et al., 2019). The mtDNA containing deletions in the liver samples were able to be detected by PCR. Notably, the level of mitochondrial DNA (mtDNA) deletion was significantly increased in the aged livers of old WT mice (Figure 5c). Conversely, in the old Cisd2TG mice, our results revealed that there is a significant decrease in the ROS/RNS and MDA levels, as well as a significant reduction in mtDNA deletion (Figure 5a–c).

A previous study has revealed that the ratio of the reduced form of glutathione (GSH) to the oxidized form of glutathione (GSSG) is able to serve as an indicator of the redox environment (Wang et al., 2020). To calculate the GSH/GSSG ratio, the amounts of glutathione of these two types in liver tissue samples were determined (Figure 5d,e). In the livers of old WT (26‐mo) mice, the GSH/GSSG ratio was significantly lower compared with that the livers of young WT (3‐mo) mice (Figure 5f). Remarkably, the GSH/GSSG ratio was significantly higher in the livers of old Cisd2TG (26‐mo) mice compared with old WT (26‐mo) mice. These results indicate that Cisd2 appears to rescue, at least in part, the aging‐induced elevation of oxidized glutathione. Moreover, in the aged liver of old WT mice, several antioxidant enzymes, namely Sod3, Nqo1, Gstm2, Gstm4, and Mgst3, have significantly increased expression levels in response to the elevated oxidative stress. Interestingly, in the old Cisd2TG mice, the expression pattern of these antioxidant enzymes was found to be similar to that in the young WT mice (Figure 5g). Taken together, these findings suggest that a high level of Cisd2 protein effectively suppresses the increase in oxidative stress associated with aging, thereby limiting lipotoxicity and attenuating mitochondrial DNA deletion.

### 2.6 Cisd2 appears to protect the liver via an autonomous effect on the hepatocytes

To study whether Cisd2 protects the liver via an autonomous regulation of hepatocyte lipid metabolism, oxidative stress, and mitochondrial function, we use the AML12 cell line as a hepatocyte cell model; this was established from the hepatocytes of a transgenic mouse (Wu et al., 1994). The AML12‐Cisd2KO cell line was generated in vitro via the use of CRISPR/Cas9‐mediated genome editing technology to disrupt both alleles of the Cisd2 gene (Figure S3a). Furthermore, these Cisd2KO cells then had the Cisd2 protein re‐expressed by a lentivirus‐mediated methodology that generated the AML12‐Cisd2RE cell line (Figure S3a); the Cisd2RE cell line was used as a control to make sure that the phenotypic defects of the Cisd2KO cell line are indeed caused by Cisd2 deficiency rather than an off‐target effect. Notably, in the AML12‐Cisd2KO hepatocytes, there is a significant increase in the level of intracellular lipids, in the level of ROS/RNS, and in the amount of lipid peroxidation (Figure S3b–d); these abnormalities affecting lipid metabolism and oxidative stress are very similar to those observed in the aged livers of the old WT mice (Figure 1g; Figure 5a,b). Moreover, in the AML12‐Cisd2KO hepatocytes, there is a significant decrease in mitochondrial respiration as monitored by the mitochondrial oxygen consumption rate (OCR) (Figure S3e). Intriguingly, in the AML12‐Cisd2RE hepatocytes, all of the metabolic defects, as well as the oxidative stress, disappear (Figure S3b–e), which indicates that Cisd2 deficiency is indeed the factor that leads to the metabolic abnormalities in the AML12‐Cisd2KO hepatocytes. Interestingly, in the AML12‐Cisd2KO hepatocytes, we also confirmed that the mRNA levels of Apoa4 and Hmox1, which are the downstream target genes of Hnf4a and Nrf2, are up‐regulated, while in the AML12‐Cisd2RE hepatocytes, the up‐regulation of these two genes is reversed (Figure S3f–i). Additionally, in mice at an age of 3 months old, fresh liver tissue samples obtained from the hepatocyte‐specific Cisd2KO mice displayed a mitochondrial phenotype that had impaired respiration as evidenced by a decrease of mitochondrial OCR. On the contrary, similar tissue samples obtained from the Cisd2TG mice show an improved trend in mitochondrial OCR compared to the controls (Figure S1b). Taken together, these findings suggest that Cisd2 has an autonomous effect in the liver on hepatocytes via the regulation of lipid metabolism, oxidative stress, and mitochondrial function.

## 3 DISCUSSION

**FIGURE 6:**
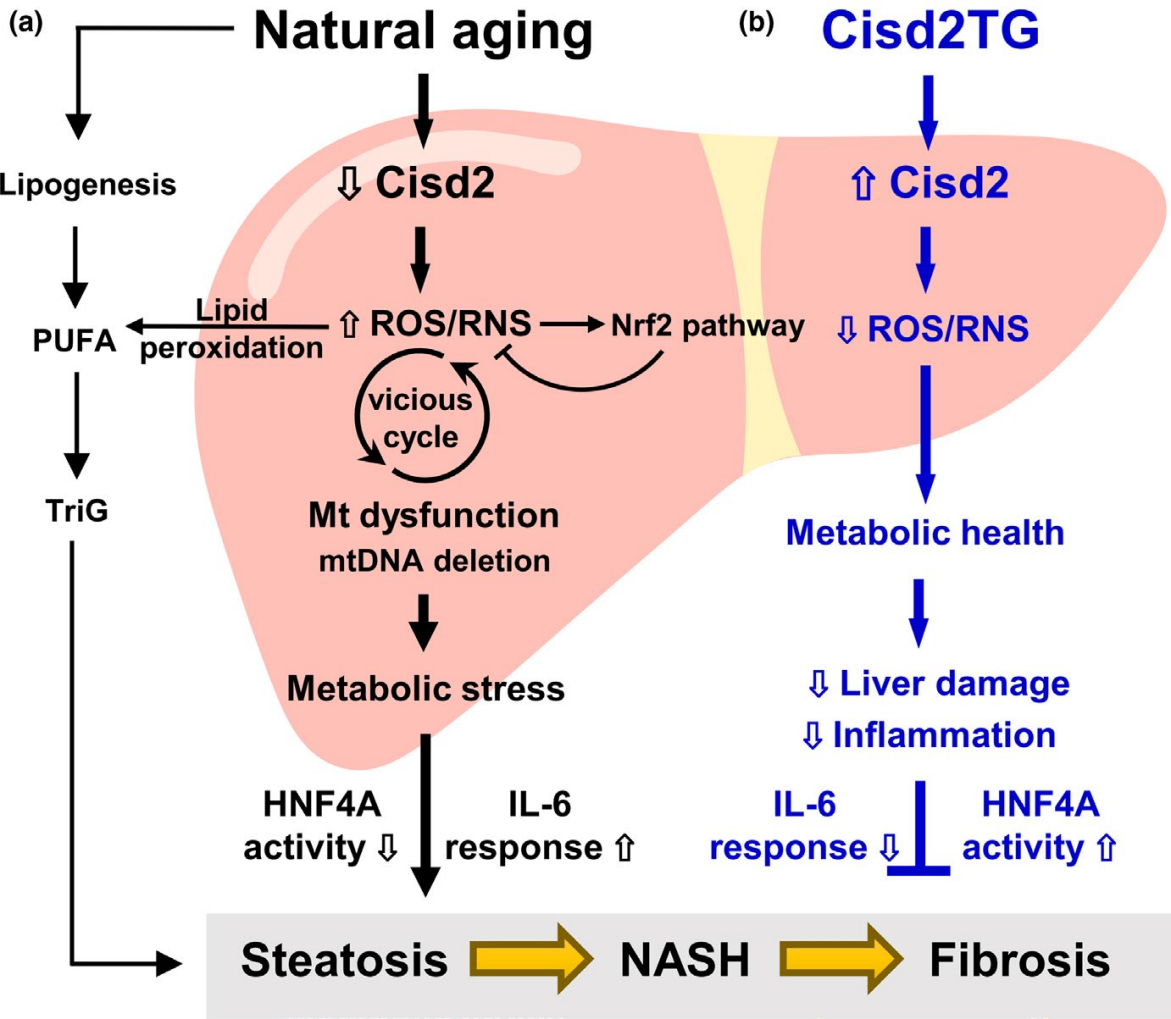
Cisd2 slows down liver aging by maintaining metabolic homeostasis and attenuating age‐related pathological damage and its associated functional decline. (a) During natural aging, the protein level of Cisd2 is significantly decreased by about 50% in the aged livers of old mice, which was associated with dysregulated lipid metabolism and fatty liver disease. Elevated levels of ROS/RNS, increased lipid peroxidation, and a higher frequency of mitochondrial DNA deletion, as well as mitochondrial dysfunction thus form a vicious cycle. Various Nrf2‐mediated mechanisms are then activated to deal with the oxidative stress. This Nrf2 pathway is likely to be a compensatory effect that occurs in response to the elevated ROS/RNS levels. Subsequently, the vicious cycle of oxidative stress and mitochondrial dysfunction is likely to lead to metabolic stress, and the disruption of various important gene networks, including the Hnf4a pathway, which is crucial to hepatocyte function, as well as the activation of a range of inflammation responses, such as the IL‐6 pathway. Overall, these changes will accelerate the development of steatosis (fatty liver disease), NASH (fatty liver disease with inflammation), and fibrosis, thereby triggering liver aging. (b) In the Cisd2TG mice, a persistently high level of Cisd2 in the liver throughout the mouse lifetime appears to maintain a youthful transcriptomic profile, thus counteracting the age‐related structural damage and related functional decline

The major finding of the present study is that a persistently high level of Cisd2 expression in the Cisd2TG mice is able to slow down liver aging, attenuate age‐related functional decline of the liver, and reduce structural damage to the liver (a graphic summary is shown in Figure 6). Several novel findings are pinpointed. Firstly, Cisd2 attenuates age‐related dysregulation of lipid metabolism and mitigates the progression of NAFLD toward NASH and fibrosis. Secondly, consistent with the pathological findings, the aged livers of old mice undergo extensive transcriptomic alterations that can be associated with steatosis, hepatitis, fibrosis, and xenobiotic detoxification, as revealed by RNA sequencing analysis. Intriguingly, a youthful transcriptomic profile, which is similar to that present in the young mice at 3 months old, was able to be observed in old Cisd2 transgenic mice at 26 months old. Thirdly, an enhanced level of Cisd2 expression suppresses age‐associated dysregulation of various transcription regulators, specifically Nrf2, IL‐6, and Hnf4a, and this maintains the transcriptional network resulting in a more normal pattern similar to that observed in young mice. Finally, a high level of Cisd2 protein appears to be able to protect the liver from oxidative stress and reduce mitochondrial DNA deletions.

### 3.1 A higher level of Cisd2 during old age results in a youthful transcriptomic profile in the livers of old mice

There are a number of known liver pathologies, including hepatic steatosis, increased infiltration of inflammatory cells (mainly Kupffer cells), fibrosis (hepatic stellate cells activation and collagen deposition), and abnormal cell death, all of which are present in the aged livers of old mice (Hunt et al., 2019). Given that an age‐dependent decrease of Cisd2 expression has been detected in various tissues during normal aging (Chen et al., 2009b, 2010; Shen et al., 2021), it is not surprising that Cisd2 is also down‐regulated in the aging liver (Figure 1a). Cisd2 haploinsufficiency in the heterozygous Cisd2 knockout (KO) mice predisposes the mice to develop NAFLD and then accelerates the carcinogenesis of HCC at a later stage (Shen et al., 2017). Therefore, down‐regulation of Cisd2 by 50% during old ages is likely to create a phenotype resembling the heterozygous Cisd2KO mice. In addition, moderate fibrosis is a prominent histological hallmark of aging in a range of organ systems, including the heart, kidney, and liver. In our study, the presence of overt steatosis, macrophage infiltration, and fibrosis in aged livers was found to be remarkably attenuated in the Cisd2TG mice (Figure 1g–j), which supports the hypothesis that maintaining a persistently high level of Cisd2 throughout a lifetime indeed is able to delay aging and result in an old mouse that has a younger and healthier liver. Conversely, a recent study has suggested that Cisd2 deficiency drives a vicious cycle of impaired mitochondrial function, disruption of Ca^2+^ homeostasis, increased ROS levels, and higher levels of ER stress (reviewed in Shen et al., 2021). Taken together, these molecular events are all likely to contribute to the development of fatty liver disease and metabolic dysfunction when Cisd2 is down‐regulated.

### 3.2 Aging is characterized by increased variation between different individuals in terms of their transcriptomic profiles

The PCA scatter plot shows that young mice cluster tightly together, while old mice show much greater individual variability (Figure 2a), which strongly implies the presence of increased variation in terms of gene expression and supports the hypothesis that this is the mechanism that brings about age‐related cellular degeneration (Bahar et al., 2006). With increasing age, we were able to note that more genes are up‐regulated than down‐regulated (Figure 2b), which is consistent with previous microarray and RNA‐seq analyses (Lee et al., 2012; White et al., 2015). If we consider that actively transcribed genes need to be tightly regulated, we can speculate that epigenetic remodeling present during liver aging (Bacalini et al., 2019) may be the potential mechanism causing universal transcriptional induction. Importantly, our findings provide evidence that alterations in the global transcriptome profile, as well as the dramatic variations in gene expression associated with aging, are reversed in 26‐mo Cisd2TG mice. This favors a prominent role for Cisd2 in liver aging.

### 3.3 Oxidative stress and mitochondrial dysfunction in the aging liver

Our results reveal that there is an elevated level of ROS/RNS, as well as a reduced GSH/GSSG ratio, in the aged livers of old WT mice (Figure 5a,f). Additionally, our previous and current studies suggest that, in the Cisd2TG mice, an enhanced level of Cisd2 maintains redox homeostasis across a variety of organs and tissues (Huang et al., 2018; Shen et al., 2017; Yeh et al., 2019). Oxidative stress is a major risk factor during aging and is associated with age‐related diseases. ROS produces harmful effects on lipids, proteins, and nucleic acids, and these result in an accumulation of lipid peroxidation products and extensive mtDNA damage. Notably, in the aging liver, previous studies have reported that there is a higher level of mtDNA oxidative damage compared with nuclear DNA oxidative damage (Hamilton et al., 2001). ROS‐induced mtDNA damage impairs mitochondrial oxidative phosphorylation and this further enhances the production of ROS, thereby establishing a vicious cycle in the aging liver. However, it would seem that Cisd2 expression at a level similar to young WT mice in the Cisd2TG mice prevents the liver from developing this vicious cycle.

### 3.4 The Nrf2‐mediated stress response

Both the IPA upstream regulator analysis and the IPA toxicity list reveal that “NFE2L2 (NRF2)” and “NRF2‐mediated oxidative stress” are among the top regulated regulators and pathways (Figures 3a and 4a). NRF2 protects against oxidative stress by controlling the expression of various cytoprotective enzymes and antioxidant proteins (Schmidlin et al., 2019). A previous study has suggested that induction of HO‐1 (HMOX1), which is one of the most important antioxidant proteins among the downstream target genes of Nrf2, has a protective effect on the liver under the stress conditions found during various liver diseases (Protzer et al., 2007; Tsui et al., 2005). In the aged liver of old WT mice, an increased ROS level was found to be accompanied by up‐regulation of Nrf2 target genes. However, in the liver of old Cisd2TG mice, the ROS level is significantly reduced; this was accompanied by a significant decrease in the expression of Nrf2 downstream target genes (Figure 4c). Thus, it seems likely that the activation of Nrf2 pathway in the aging liver is an adaptive response that protects against increased oxidative stress.

### 3.5 Lipid metabolism in the aging liver

The present study has also elucidated how Cisd2 acts as a protective mechanism against age‐related fatty liver and dysregulation of lipid metabolism. In the Cisd2TG mice, a persistently high level of Cisd2 is able to suppress the age‐related induction of various lipid metabolism genes, including Scd1 (a lipogenic enzyme), G0S2 (a lipolysis inhibitor), Cd36 (a fatty acid transporter), Nrip1 (RIP140), and Id1 (a transcription corepressor), as shown in Figure 3. Up‐regulation of these lipid metabolism genes in the aged liver seems to result in an increase in de novo lipogenesis, which then leads to fat accumulation in the liver. Interesting, an increase in Ppargc1a (PGC‐1α) expression was also found in the aged liver of old mice (Figure 3c). In addition to this finding, a number of age‐dependent changes in various upstream transcription regulators were predicted, and, among these, the transcription regulator PPARα (Table S1) was clearly identified as a strong candidate. Increased expression of PGC‐1α and PPARα has been reported to increase fatty acid oxidative metabolism (Cheng et al., 2018). Whether the increase in PGC‐1α expression and the activation of PPARα are compensatory effects in response to the lipid accumulation in the aged liver remain unclear and deserves further investigation in the future.

## 4 CONCLUSION

The liver plays a pivotal role in the aging of mammals, and this seems to occur via the modulation of a variety of metabolism and detoxification pathways. The present study provides genetic evidence demonstrating that Cisd2 is a promising target for slowing down liver aging. Since Cisd2 is down‐regulated in the liver during natural aging, the up‐regulation of Cisd2 expression by a Cisd2 activator should help to protect the liver from age‐related structural damage and the associated functional decline. We anticipate that development of therapeutic agents that bring about an effective enhancement of Cisd2 expression will promote longevity and improve healthspan. However, the potential causal roles of the Hnf4a, IL‐6, and Nrf2 signal pathways in Cisd2‐mediated longevity remained to be explored.

## 5 EXPERIMENTAL PROCEDURES

### 5.1 Mice

The Cisd2 BAC transgenic (Cisd2TG) mice were generated on a C57BL/6 background (Shen et al., 2017; Wu et al., 2012). The Cisd2TG mice carry four copies of Cisd2 genes, namely two endogenous Cisd2 alleles and two transgenic copies of the Cisd2 BAC. Since the prevalence of NAFLD in males is significantly higher than in females at all ages (Lonardo et al., 2019), male mice were used for all experiments in this study. The mice were maintained at 21 ± 1°C under a regular 12/12 h light/dark cycle and fed a normal chow diet ad libitum. When assessing age‐dependent hepatic steatosis, the experiments were performed using wild‐type mice aged 3 and 26 months old. Additionally, Cisd2TG mice were sacrificed at 26 months old. The body weights and liver weights were recorded at sacrifice. The serum concentrations of alanine aminotransferase (ALT), aspartate aminotransferase (AST), and alkaline phosphatase (ALP) of the mice were determined using a DRI‐CHEM 3500s (FUJIFILM). All animal protocols were approved by the Institutional Animal Care and Use Committee (IACUC) of National Yang Ming Chiao Tung University (No. 1080410).

### 5.2 Liver histopathology

Liver tissue samples were harvested and then fixed in 10% buffered formalin solution for paraffin embedding. Hematoxylin and eosin (H&E) staining and Masson's trichrome staining (Muto Pure Chemicals) of 3 μm thickness liver sections were carried out using standard protocols. Areas of fibrosis and the total area investigated were determined by Image J software from a number of image fields obtaining from each slide.

### 5.3 Immunohistochemistry

Immunohistochemistry (IHC) staining was carried out on paraffin‐embedded liver sections (3 μm) using antibodies against F4/80 (123101, BioLegend) and against Ki67 (550609, BD Pharmingen) separately, and this was followed by counterstaining with hematoxylin. Briefly, sections were deparaffinized and rehydrated, and they then underwent antigen‐retrieved using target retrieval solution (S1699, Dako). Next, the liver sections were treated with 3% H_2_O_2_ in PBS, which was followed by blocking with 5% bovine serum albumin solution. After incubation with each primary antibody in antibody diluent (ab64211, Abcam), the signals were detected by the Labeled Streptavidin–Biotin (LSAB) staining method (K0679, Dako).

### 5.4 Western blotting

Liver tissues from each mouse from the three different groups were homogenized in lysis buffer (50 mM Tris at pH 7.4, 100 mM NaCl, 1 mM EDTA, and 1% Triton X‐100 in the presence of complete protease inhibitor and phosphatase inhibitor cocktails (Roche). The hepatic lysates (10 μg proteins) were separated by 12% SDS‐PAGE and transferred onto polyvinylidene fluoride (PVDF) membrane. The membranes were blocked using 5% (w/v) non‐fat freeze‐dried milk in buffer, then probed with anti‐Cisd2 (Chen et al., 2009a) or anti‐Gapdh (MAB374, Millipore) antibody, and finally developed using ECL reagents (34580, Thermo). Quantitative densitometric analysis using Image J software was performed.

### 5.5 Liver TriG, MDA, ROS/RNS, and GSH/GSSG levels

Liver triglyceride (TriG) content was extracted by homogenization of each liver tissue sample with a mixture of 1:2 chloroform and methanol (v/v) as described previously (Shen et al., 2017). The hepatic content of TriG was determined using a TriG assay kit (TR0100, Sigma). Hepatic levels of lipid peroxidation products (malondialdehyde, MDA) were determined by thiobarbituric acid reactive substances (TBARS) assay using a commercially available kit (STA‐330, Cell Biolabs). Quantification of total reactive oxygen species (ROS) and reactive nitrogen species (RNS) present in the liver tissue was carried out using a specific ROS/RNS probe, namely dichlorodihydrofluorescein DiOxyQ (DCFH‐DiOxyQ), through the use of a OxiSelect in vitro ROS/RNS assay kit (STA‐347, Cell Biolabs). The levels were normalized to total protein amount of liver tissues. The levels of GSH and GSSG were measured using a GSH/GSSG Ratio Detection Assay Kit II (ab205811, Abcam).

### 5.6 Presence of mitochondrial DNA (mtDNA) deletions

Detection and quantification of DNA deletions in mitochondrial genome were performed by PCR analysis of genomic DNA extracted from the liver (Tanhauser & Laipis, 1995). In brief, specific deletions in the mtDNA flanked by known direct repeat sequences (D‐1 or D‐17) were amplified using a short PCR cycle. The D‐1 primers were able to amplify either a 748‐bp mtDNA deletion product or a full‐length 4,615‐bp undeleted mtDNA product (mtDNA^WT^). The D‐17 primers were able to amplify either a 851‐bp mtDNA deletion product or a full‐length 4,672‐bp undeleted mtDNA product (mtDNA^WT^).

### 5.7 RNA extraction, sequencing, and analysis

Total RNA extraction from the mouse liver tissue samples was performed using TRI Reagent (T9424, Sigma) using the phenol/chloroform method. The library preparation and sequencing were performed by National Yang‐Ming University VYM Genome Research Center. The analysis generated a sequencing depth of at least 20 million reads for each sample by single‐end sequencing. Transcript abundance was measured as transcripts per million (TPM). A total of 11,000–12,000 genes were retained after filtering for expressed genes (minimal count of TPM >1 detected in at least 50% samples). The resulting *p* values from Student's t test were adjusted using the Benjamini–Hochberg method. Differentially expressed genes (DEGs) were identified using cutoff thresholds for the false discovery rate (FDR) and using a fold change, as indicated in the legend of each figure.

### 5.8 Functional analysis of differentially expressed genes

Gene ontology annotation of the DEGs was carried out using the statistical overrepresentation test from PANTHER (http://pantherdb.org/) with GO‐Slim Biological Process. The top ten significant processes with a fold enrichment of >4.5 and an FDR of <5% are presented in the GOslim analysis. Upstream regulator analysis and the Ingenuity Tox List tool were used to the identified genes via IPA (Ingenuity^®^Systems, http://www.ingenuity.com). The top ontologies that are able to give relevant biological insights related to the liver are presented.

### 5.9 Statistical analysis

All data are presented as mean ± SD. Unpaired two‐tailed Student's *t* test was used to compare two groups. Significant differences were defined as having a *p * < 0.05. The RNA‐seq dataset from the TPM was loaded into the EZinfo 3.0.3 software (Umetrics) package for principal component analysis (PCA). TPM values were transformed into *z*‐scores and these were used to create heatmaps, which were in turn generated by Multi Experiment Viewer (MEV) 4.9 software (Saeed et al., 2003).

## CONFLICT OF INTEREST

The authors declare no conflict of interest.

## AUTHOR CONTRIBUTIONS

CHL and TFT designed the study concept. ZQS performed the experiments. ZQS and YLH analyzed the data with assistance from CHH, YLH, and ZQS drafted the manuscript. TFT and CHL edited and revised the final version of the manuscript.

## Supporting information

Supplementary Material

## Data Availability

The data that support the findings of this study are available from the corresponding author upon reasonable request.

## References

[acel13523-bib-0001] Anstee, Q. M. , Reeves, H. L. , Kotsiliti, E. , Govaere, O. , & Heikenwalder, M. (2019). From NASH to HCC: Current concepts and future challenges. Nature Reviews: Gastroenterology & Hepatology, 16(7), 411–428. 10.1038/s41575-019-0145-7 31028350

[acel13523-bib-0002] Bacalini, M. G. , Franceschi, C. , Gentilini, D. , Ravaioli, F. , Zhou, X. , Remondini, D. , Pirazzini, C. , Giuliani, C. , Marasco, E. , Gensous, N. , Di Blasio, A. M. , Ellis, E. , Gramignoli, R. , Castellani, G. , Capri, M. , Strom, S. , Nardini, C. , Cescon, M. , Grazi, G. L. , … Garagnani, P. (2019). Molecular aging of human liver: An epigenetic/transcriptomic signature. Journals of Gerontology. Series A: Biological Sciences and Medical Sciences, 74(1), 1–8. 10.1093/gerona/gly048 29554203

[acel13523-bib-0003] Bahar, R. , Hartmann, C. H. , Rodriguez, K. A. , Denny, A. D. , Busuttil, R. A. , Dolle, M. E. , Calder, R. B. , Chisholm, G. B. , Pollock, B. H. , Klein, C. A. , & Vijg, J. (2006). Increased cell‐to‐cell variation in gene expression in ageing mouse heart. Nature, 441(7096), 1011–1014. 10.1038/nature04844 16791200

[acel13523-bib-0004] Chakravarthy, M. V. , & Neuschwander‐Tetri, B. A. (2020). The metabolic basis of nonalcoholic steatohepatitis. Endocrinology, Diabetes & Metabolism, 3(4), e00112. 10.1002/edm2.112 PMC7576253 33102794

[acel13523-bib-0005] Chen, Y. F. , Kao, C. H. , Chen, Y. T. , Wang, C. H. , Wu, C. Y. , Tsai, C. Y. , Liu, F. C. , Yang, C. W. , Wei, Y. H. , Hsu, M. T. , Tsai, S. F. , & Tsai, T. F. (2009a). Cisd2 deficiency drives premature aging and causes mitochondria‐mediated defects in mice. Genes and Development, 23(10), 1183–1194. 10.1101/gad.1779509 19451219 PMC2685531

[acel13523-bib-0006] Chen, Y. F. , Kao, C. H. , Kirby, R. , & Tsai, T. F. (2009b). Cisd2 mediates mitochondrial integrity and life span in mammals. Autophagy, 5(7), 1043–1045. 10.4161/auto.5.7.9351 19717971

[acel13523-bib-0007] Chen, Y. F. , Wu, C. Y. , Kirby, R. , Kao, C. H. , & Tsai, T. F. (2010). A role for the CISD2 gene in lifespan control and human disease. Annals of the New York Academy of Sciences, 1201, 58–64. 10.1111/j.1749-6632.2010.05619.x 20649540

[acel13523-bib-0008] Chen, Z. , Tian, R. , She, Z. , Cai, J. , & Li, H. (2020). Role of oxidative stress in the pathogenesis of nonalcoholic fatty liver disease. Free Radical Biology and Medicine, 152, 116–141. 10.1016/j.freeradbiomed.2020.02.025 32156524

[acel13523-bib-0009] Cheng, C. F. , Ku, H. C. , & Lin, H. (2018). PGC‐1alpha as a pivotal factor in lipid and metabolic regulation. International Journal of Molecular Sciences, 19(11), 10.3390/ijms19113447 PMC6274980 30400212

[acel13523-bib-0010] Chocron, E. S. , Munkacsy, E. , & Pickering, A. M. (2019). Cause or casualty: The role of mitochondrial DNA in aging and age‐associated disease. Biochimica et Biophysica Acta (BBA)—Molecular Basis of Disease, 1865(2), 285–297. 10.1016/j.bbadis.2018.09.035 30419337 PMC6310633

[acel13523-bib-0011] Hamilton, M. L. , Van Remmen, H. , Drake, J. A. , Yang, H. , Guo, Z. M. , Kewitt, K. , Walter, C. A. , & Richardson, A. (2001). Does oxidative damage to DNA increase with age? Proceedings of the National Academy of Sciences of the United States of America, 98(18), 10469–10474. doi:10.1073/pnas.171202698 11517304 PMC56984

[acel13523-bib-0012] Huang, Y. L. , Shen, Z. Q. , Wu, C. Y. , Teng, Y. C. , Liao, C. C. , Kao, C. H. , Chen, L. K. , Lin, C. H. , & Tsai, T. F. (2018). Comparative proteomic profiling reveals a role for Cisd2 in skeletal muscle aging. Aging Cell, 17(1), 10.1111/acel.12705 PMC5770874 29168286

[acel13523-bib-0013] Hunt, N. J. , Kang, S. W. S. , Lockwood, G. P. , Le Couteur, D. G. , & Cogger, V. C. (2019). Hallmarks of aging in the liver. Computational and Structural Biotechnology Journal, 17, 1151–1161. 10.1016/j.csbj.2019.07.021 31462971 PMC6709368

[acel13523-bib-0014] Lee, J. S. , Ward, W. O. , Ren, H. , Vallanat, B. , Darlington, G. J. , Han, E. S. , Laguna, J. C. , DeFord, J. H. , Papaconstantinou, J. , Selman, C. , & Corton, J. C. (2012). Meta‐analysis of gene expression in the mouse liver reveals biomarkers associated with inflammation increased early during aging. Mechanisms of Ageing and Development, 133(7), 467–478. 10.1016/j.mad.2012.05.006 22704917

[acel13523-bib-0015] Lonardo, A. , Nascimbeni, F. , Ballestri, S. , Fairweather, D. , Win, S. , Than, T. A. , Abdelmalek, M. F. , & Suzuki, A. (2019). Sex differences in nonalcoholic fatty liver disease: State of the art and identification of research gaps. Hepatology, 70(4), 1457–1469. 10.1002/hep.30626 30924946 PMC6766425

[acel13523-bib-0016] Lu, H. (2016). Crosstalk of HNF4alpha with extracellular and intracellular signaling pathways in the regulation of hepatic metabolism of drugs and lipids. Acta Pharmaceutica Sinica B, 6(5), 393–408. 10.1016/j.apsb.2016.07.003 27709008 PMC5045537

[acel13523-bib-0017] Marjot, T. , Moolla, A. , Cobbold, J. F. , Hodson, L. , & Tomlinson, J. W. (2020). Nonalcoholic fatty liver disease in adults: Current concepts in etiology, outcomes, and management. Endocrine Reviews, 41(1), 10.1210/endrev/bnz009 31629366

[acel13523-bib-0018] Masarone, M. , Rosato, V. , Dallio, M. , Gravina, A. G. , Aglitti, A. , Loguercio, C. , Federico, A. , & Persico, M. (2018). Role of oxidative stress in pathophysiology of nonalcoholic fatty liver disease. Oxidative Medicine and Cellular Longevity, 2018, 1–14. 10.1155/2018/9547613 PMC6016172 29991976

[acel13523-bib-0019] Nissanka, N. , Minczuk, M. , & Moraes, C. T. (2019). Mechanisms of mitochondrial DNA deletion formation. Trends in Genetics, 35(3), 235–244. 10.1016/j.tig.2019.01.001 30691869

[acel13523-bib-0020] Protzer, U. , Seyfried, S. , Quasdorff, M. , Sass, G. , Svorcova, M. , Webb, D. , Bohne, F. , Hosel, M. , Schirmacher, P. , & Tiegs, G. (2007). Antiviral activity and hepatoprotection by heme oxygenase‐1 in hepatitis B virus infection. Gastroenterology, 133(4), 1156–1165. 10.1053/j.gastro.2007.07.021 17919491

[acel13523-bib-0021] Saeed, A. I. , Sharov, V. , White, J. , Li, J. , Liang, W. , Bhagabati, N. , Braisted, J. , Klapa, M. , Currier, T. , Thiagarajan, M. , Sturn, A. , Snuffin, M. , Rezantsev, A. , Popov, D. , Ryltsov, A. , Kostukovich, E. , Borisovsky, I. , Liu, Z. , Vinsavich, A. , … Quackenbush, J. (2003). TM4: A free, open‐source system for microarray data management and analysis. BioTechniques, 34(2), 374–378. 10.2144/03342mt01 12613259

[acel13523-bib-0022] Schmidlin, C. J. , Dodson, M. B. , Madhavan, L. , & Zhang, D. D. (2019). Redox regulation by NRF2 in aging and disease. Free Radical Biology and Medicine, 134, 702–707. 10.1016/j.freeradbiomed.2019.01.016 30654017 PMC6588470

[acel13523-bib-0023] Shen, Z. Q. , Chen, Y. F. , Chen, J. R. , Jou, Y. S. , Wu, P. C. , Kao, C. H. , Wang, C. H. , Huang, Y. L. , Chen, C. F. , Huang, T. S. , Shyu, Y. C. , Tsai, S. F. , Kao, L. S. , & Tsai, T. F. (2017). CISD2 haploinsufficiency disrupts calcium homeostasis, causes nonalcoholic fatty liver disease, and promotes hepatocellular carcinoma. Cell Reports, 21(8), 2198–2211. 10.1016/j.celrep.2017.10.099 29166610

[acel13523-bib-0024] Shen, Z. Q. , Huang, Y. L. , Teng, Y. C. , Wang, T. W. , Kao, C. H. , Yeh, C. H. , & Tsai, T. F. (2021). CISD2 maintains cellular homeostasis. Biochimica et Biophysica Acta (BBA)—Molecular Cell Research, 1868(4), 118954. 10.1016/j.bbamcr.2021.118954 33422617

[acel13523-bib-0025] Shen, Z. Q. , Huang, Y. L. , & Tsai, T. F. (2018). Cisd2 haploinsufficiency: A driving force for hepatocellular carcinoma. Molecular & Cellular Oncology, 5(3), e1441627. 10.1080/23723556.2018.1441627 30250893 PMC6149959

[acel13523-bib-0026] Tanhauser, S. M. , & Laipis, P. J. (1995). Multiple deletions are detectable in mitochondrial DNA of aging mice. Journal of Biological Chemistry, 270(42), 24769–24775. 10.1074/jbc.270.42.24769 7559594

[acel13523-bib-0027] Tilg, H. , & Moschen, A. R. (2010). Evolution of inflammation in nonalcoholic fatty liver disease: The multiple parallel hits hypothesis. Hepatology, 52(5), 1836–1846. 10.1002/hep.24001 21038418

[acel13523-bib-0028] Tsui, T. Y. , Lau, C. K. , Ma, J. , Wu, X. , Wang, Y. Q. , Farkas, S. , Xu, R. , Schlitt, H. J. , & Fan, S. T. (2005). rAAV‐mediated stable expression of heme oxygenase‐1 in stellate cells: A new approach to attenuate liver fibrosis in rats. Hepatology, 42(2), 335–342. 10.1002/hep.20803 16025519

[acel13523-bib-0029] Wang, C. H. , Kao, C. H. , Chen, Y. F. , Wei, Y. H. , & Tsai, T. F. (2014). Cisd2 mediates lifespan: Is there an interconnection among Ca(2)(+) homeostasis, autophagy, and lifespan? Free Radical Research, 48(9), 1109–1114. 10.3109/10715762.2014.936431 24974737

[acel13523-bib-0030] Wang, L. , Ahn, Y. J. , & Asmis, R. (2020). Sexual dimorphism in glutathione metabolism and glutathione‐dependent responses. Redox Biology, 31, 10.1016/j.redox.2019.101410. 101410 PMC7212491 31883838

[acel13523-bib-0031] White, R. R. , Milholland, B. , MacRae, S. L. , Lin, M. , Zheng, D. , & Vijg, J. (2015). Comprehensive transcriptional landscape of aging mouse liver. BMC Genomics, 16, 899. 10.1186/s12864-015-2061-8 26541291 PMC4636074

[acel13523-bib-0032] Wu, C. Y. , Chen, Y. F. , Wang, C. H. , Kao, C. H. , Zhuang, H. W. , Chen, C. C. , Chen, L. K. , Kirby, R. , Wei, Y. H. , Tsai, S. F. , & Tsai, T. F. (2012). A persistent level of Cisd2 extends healthy lifespan and delays aging in mice. Human Molecular Genetics, 21(18), 3956–3968. 10.1093/hmg/dds210 22661501

[acel13523-bib-0033] Wu, J. C. , Merlino, G. , & Fausto, N. (1994). Establishment and characterization of differentiated, nontransformed hepatocyte cell lines derived from mice transgenic for transforming growth factor alpha. Proceedings of the National Academy of Sciences of the United States of America, 91(2), 674–678. 10.1073/pnas.91.2.674 7904757 PMC43011

[acel13523-bib-0034] Yeh, C. H. , Chou, Y. J. , Kao, C. H. , & Tsai, T. F. (2020). Mitochondria and calcium homeostasis: Cisd2 as a big player in cardiac ageing. International Journal of Molecular Sciences, 21(23), 9238. 10.3390/ijms21239238 PMC7731030 33287440

[acel13523-bib-0035] Yeh, C. H. , Shen, Z. Q. , Hsiung, S. Y. , Wu, P. C. , Teng, Y. C. , Chou, Y. J. , Fang, S. W. , Chen, C. F. , Yan, Y. T. , Kao, L. S. , Kao, C. H. , & Tsai, T. F. (2019). Cisd2 is essential to delaying cardiac aging and to maintaining heart functions. PLoS Biology, 17(10), e3000508. 10.1371/journal.pbio.3000508 31593566 PMC6799937

