# Supplementary material for: Cisd2 slows down liver aging and attenuates age‐related metabolic dysfunction in male mice
Source: Aging Cell. 2021 Nov 22;20(12):e13523. doi: 10.1111/acel.13523 (PMC8672792; doi:10.1111/acel.13523)
Supplement: Supplementary file 1 — Supplementary Material [file ACEL-20-e13523-s001.pdf]

Extended Experimental Procedures

Cell culture

Murine hepatocyte cell line AML12 (ATCC CRL-2254) were maintained in AML12 complete medium (DMEM/F12 [11330-032, Gibco] supplemented with 10% fetal bovine serum, glutamine/penicillin/ streptomycin, 1% NEAA, 1% insulin-transferrin-selenium (ITS, 51500-056, Gibco) and 40 ng/ml dexamethasone (D2915, Sigma). The Cisd2 gene was disrupted by CRISPR/Cas9 system. The gRNA/Cas9n (D10A) plasmids were kindly provided by Dr. Tsai-Yu Tzeng, Cancer Progression Research Center-Genome Editing Core Facility, NYCU. The plasmids were co-transfected into AML12 cells by PolyJet™ reagent (SL100688, SignaGen Laboratories). After selection, deletion of Cisd2 genes was accessed by PCR amplification and DNA sequencing. Western blot analysis also confirmed the absence of Cisd2.

For generation of Cisd2 re-expression AML12 (Cisd2RE), one clone of Cisd2KO cells was subjected to lentiviral vector infection. Preparation and production of the lentivirus were conducted by the National RNAi Core Facility at Academia Sinica in Taiwan. AML12 cells were infected with lentivirus (8 µg/ml polybrene). After infection, cells were selected in 1 µg/ml puromycin.

Mitochondrial oxygen consumption

An XF^{e}24 analyzer (Seahorse Bioscience, MA) was employed to access mitochondrial respiration activities by measuring the oxygen consumption rate (OCR). After plating 4×10^4 cells/well in a XF24 V7 microplate for 12–16 h, growth medium by fresh AML12 assay medium (Sodium bicarbonate-free DMEM/F12 [12500, Gibco] supplemented with 2% FBS, 2 mM glutamine, 100 U/ml penicillin, 100 µg/ml streptomycin, 1% NEAA, 1% insulin-transferrin-selenium [ITS, 51500-056, Gibco) and 40 ng/ml dexamethasone [D2915, Sigma], pH=7.4) at 1 hr before analysis. The OCR was monitored after sequential addition of oligomycin A (1 µM), FCCP (1 µM) and rotenone (0.5 µM) with antimycin A (0.5 µM) at 37°C. The data were normalized to total protein content.

Intracellular lipids, reactive oxygen species and lipid peroxidation products

The intracellular lipids of the AML12 were stained with Oil red-O as described previously (Wang et al., 2014). To quantify the intracellular lipid amount, the Oil red-O stain was eluted from the AML12 and the absorbance at 510 nm was measured on a spectrophotometer. Absorbance reading of Oil red-O was normalized by cell numbers. Intracellular ROS/RNS and MDA levels were measured by using the same kits as for hepatic ROS/RNS and MDA, and the levels were normalized to total protein.

Real-time quantitative PCR (qPCR)

Total RNA was extracted from AML12 cells using TRI Reagent (T9424, Sigma). The cDNA was synthesized by SuperScript™ III reverse transcriptase (18080, Invitrogen) using random hexamers according to the manufacturer's instructions. The quantitative PCR (qPCR) was performed with Fast SYBR Green Master Mix (Thermo Fisher Scientific) following manufacture's standard protocol. The data were calculated using $2^{-\Delta CT}$ and normalized to Gapdh.

References

Wang, C. H., Chen, Y. F., Wu, C. Y., Wu, P. C., Huang, Y. L., Kao, C. H., . . . Wei, Y. H. (2014). Cisd2 modulates the differentiation and functioning of adipocytes by regulating intracellular Ca^{2+} homeostasis. Hum Mol Genet, 23(18), 4770-4785. doi:10.1093/hmg/ddu193

Shen, Z. Q., Chen, Y. F., Chen, J. R., Jou, Y. S., Wu, P. C., Kao, C. H., . . . Tsai, T. F. (2017). CISD2 Haploinsufficiency Disrupts Calcium Homeostasis, Causes Nonalcoholic Fatty Liver Disease, and Promotes Hepatocellular Carcinoma. Cell Rep, 21(8), 2198-2211. doi:10.1016/j.celrep.2017.10.099

Figure S1

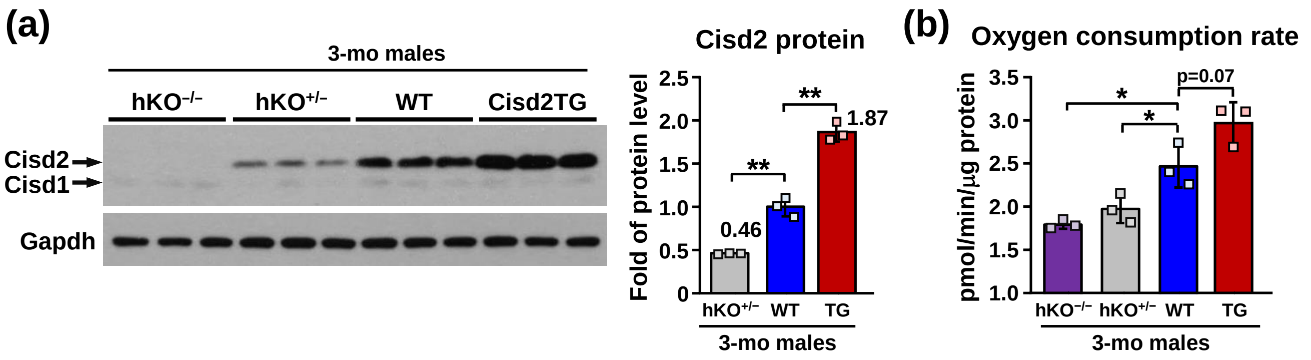

Figure S1. Dose-dependent effect of Cisd2 protein levels on the basal oxygen consumption rate in the livers of young adult mice. (a) Immunoblotting of Cisd2 protein in the livers of hepatocyte-specific Cisd2 knockout (Cisd2hKO$^{-/-}$), heterozygous hepatocyte-specific Cisd2 knockout (Cisd2hKO$^{+/-}$), WT and Cisd2TG mice at 3-month old (n = 3). **(b)** Oxygen consumption rate in the fresh liver tissues of Cisd2hKO$^{-/-}$, Cisd2hKO$^{+/-}$, WT and Cisd2TG mice at 3-month old (n = 3). Data are presented as mean ± SD. *$p < 0.05$; **$p < 0.005$. Albumin-Cre transgenic (Alb-Cre, JAX003574) mice were bred with the Cisd2f/f mice to generate Cisd2hKO$^{-/-}$ (Cisd2f/f;Alb-Cre) mice as previously described (Shen et al., 2017).

Figure S2

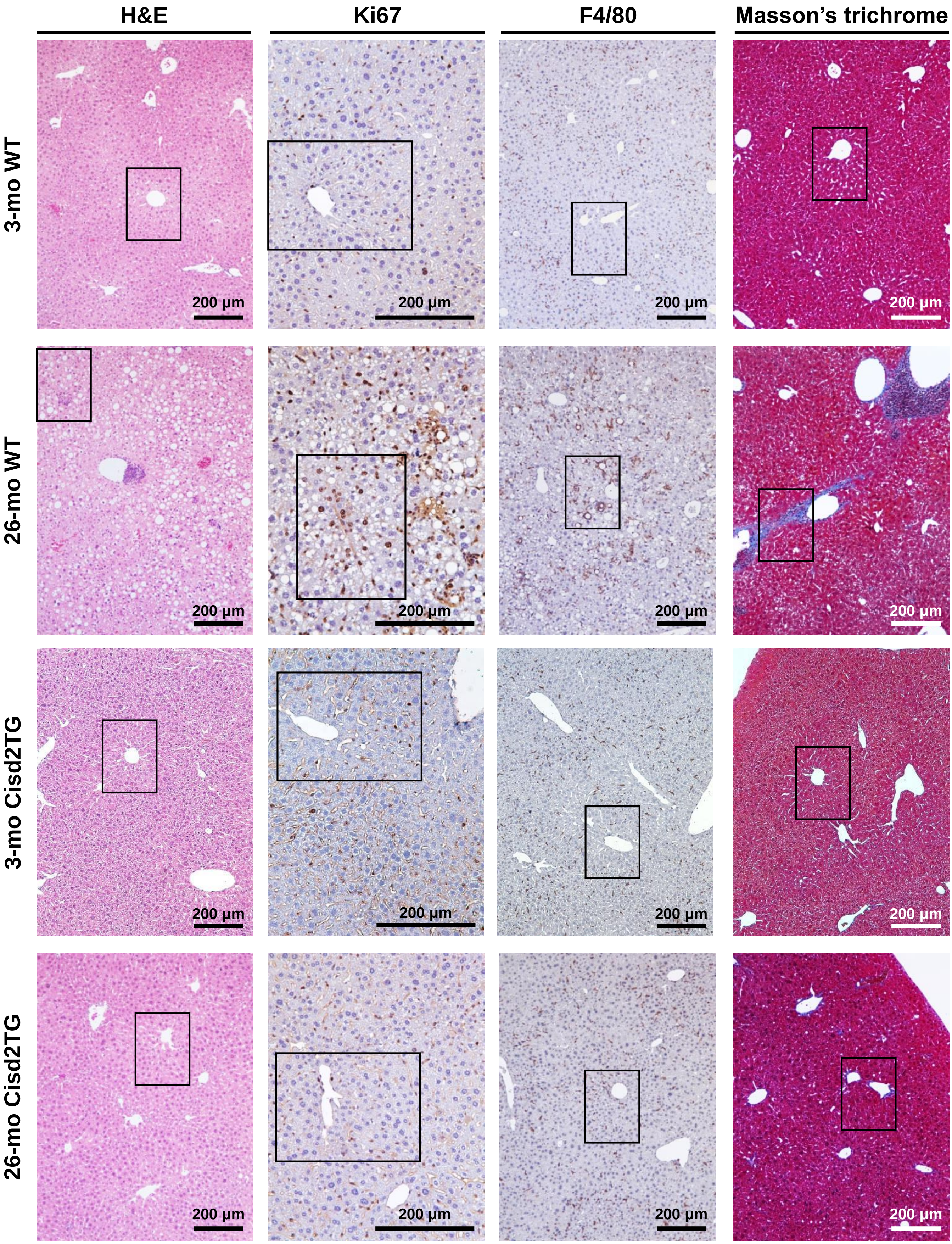

Figure S2. Histological microphotographs at low magnification in hepatic tissues from the young and old WT and Cids2TG mice. Rectangular areas are enlarged and displayed in Figure 1g. Scale bar, 200 μm.

Figure S3

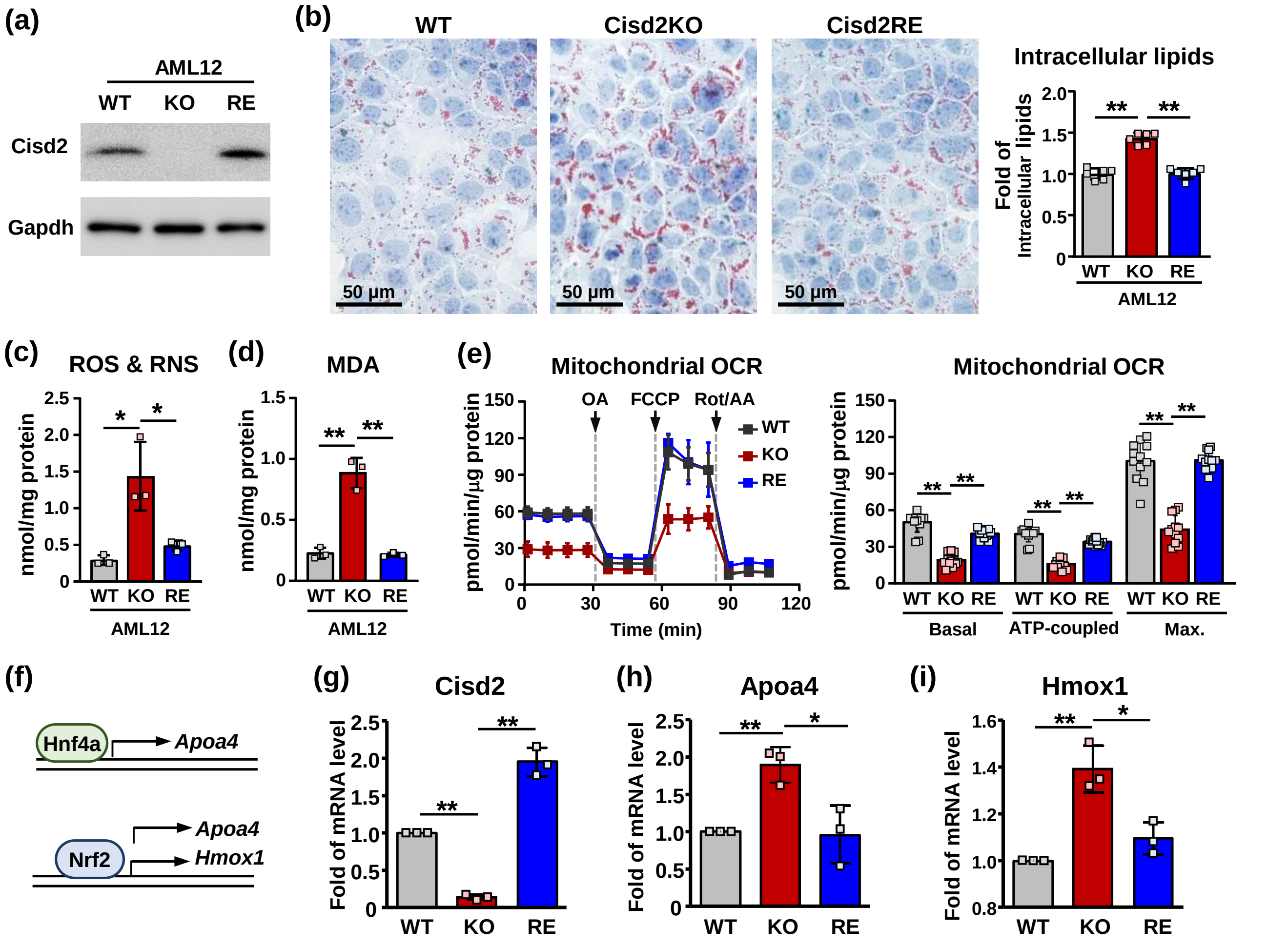

Figure S3. Cisd2 deficiency leads to lipid accumulation, mitochondrial dysfunction and oxidative stress in the AML12 mouse hepatocyte cell line. (a) Immunoblotting of Cisd2 protein in the AML12 cells that are WT, Cisd2KO, or Cisd2KO with re-expression of Cisd2 (Cisd2RE) backgrounds. **(b)** Representative micrographs of the Oil Red O staining, together with the quantification of lipid droplets, within the AML12 cells carrying the WT, Cisd2KO and Cisd2RE backgrounds. The red spots/particles are lipid droplets (primarily triglyceride) that have accumulated in the cytoplasm (n = 6). **(c)** The total levels of reactive oxygen and nitrogen species (ROS/RNS) in AML12 cells with the WT, Cisd2KO and Cisd2RE backgrounds (n = 3 for each group). **(d)** The levels of malondialdehyde (MDA, a marker of lipid peroxidation) in AML12 cells with the WT, Cisd2KO and Cisd2RE backgrounds (n = 3 for each group). **(e)** Mitochondrial oxygen consumption rates (OCRs) measured by Seahorse $XF24^e$ analyzer of AML12 cells with the WT, Cisd2KO and Cisd2RE backgrounds (n = 12 for each group). OA, oligomycin A; FCCP, carbonilcyanide p-trifluoromethoxyphenylhydrazone; Rot/AA, rotenone/antimycin A. **(f)** An outline of the representative target genes that are transcriptionally regulated by Nrf2 and Hnf4a. **(g)** The levels of Cisd2 mRNA in WT, Cisd2KO and Cisd2RE AML12 cells (n = 3). **(h)** The levels of Apoa4 mRNA in the WT, Cisd2KO and Cisd2RE AML12 cells (n = 3). **(i)** The levels of Hmox1 mRNA in the WT, Cisd2KO and Cisd2RE AML12 cells (n = 3). The mRNA expression levels were analyzed by real-time quantitative RT-PCR. All of these quantitative results were obtained from at least three independent experiments. Data are presented as mean ± SD. *$p < 0.05$; **$p < 0.005$.

Table S1. Age-dependent changes in the upstream transcription regulators and their downstream target genes.

Upstream Regulator	Molecule Type	Predicted Activation State	Activation z-score	p-value of overlap	Target molecules in dataset
TRIM24	transcription regulator	Inhibited	−4.861	8.54E−18	Bst2, CMPK2, CXCL10, DDX60, DHX58, Ifi47, IFIH1, IFIT1B, IFIT3, IRF7, IRF9, LGALS3BP, Ly6a (includes others), Ms4a4c (includes others), NMI, PHF11, PLAC8, SAMD9L, SHISA5, STAT1, TAP1, TGFBI, TLR2, USP18
**HNF4A**	transcription regulator	Inhibited	−3.852	0.00203	ABCD2, ACOT1, CAT, CCND1, CP, CYP3A5, FMO1, GATA6, Hamp/Hamp2, HLA-A, HPX, Hsd3b4 (includes others), ITGA9, MGST1, MSN, NTRK2, PSAP, RAP2B, RSAD2, SCD, Slco1a1, TF, TOX, TRIM24
DNASE2	enzyme	Inhibited	−2.804	4.3E−10	CP, CXCL10, DHX58, Hamp/Hamp2, IFIT1B, IFIT3, IRF7, Ly6a (includes others), Ms4a4c (includes others), RSAD2, TNFSF10, USP18
TXNRD1	enzyme	Inhibited	−2.36	0.000277	CBR1, CD36, GSTM1, GSTM4, HMOX1, MGST3, NQO1, SCD, SLC2A1
GSR	enzyme	Inhibited	−2.333	0.000277	CBR1, CD36, GSTM1, GSTM4, HMOX1, MGST3, NQO1, SCD, SLC2A1
BCAP31	transporter	Inhibited	−2.236	0.0186	ACACA, ADGRE1, CXCL10, SAA1, SCD
CYB5R4	enzyme	Inhibited	−2.207	0.00549	CD36, HMOX1, PPARGC1A, SCD, Scd2
MYD88	other	Activated	2	0.0138	CD14, CXCL10, SAA1, USP18
**IL6**	cytokine	Activated	2.214	0.000116	ADGRE1, APCS, CCND1, CYP2C8, CYP3A5, Hamp/Hamp2, JUN, Orm1 (includes others), SAA1, Slco1a1
TNK1	kinase	Activated	2.63	8.27E−06	IFI16, IFIH1, IFIT1B, IRF7, OAS2, TLR8, TNFSF10
**NFE2L2 (Nrf2)**	transcription regulator	Activated	3.157	2.36E−05	ACSL5, ACTG1, ALAS2, APCS, APOA4, CAT, CBR1, CELA1, CTSD, CYP4A22, DYNLL1, FMO1, GBE1, GHR, GSTM1, GSTM4, GSTP1, HMOX1, HSD3B1, HSP90AA1, HTATIP2, INMT, LY6E, MGST1, MGST3, NAT8B, NQO1, PPARGC1A, SAA1, SERPINA3, UGT2B28
PPARA	ligand-dependent nuclear receptor	Activated	3.19	1.18E-08	AADAC, ABCD2, ACACA, ACSL5, APCS, APOA4, C1QA, C1QB, C8A, C9, CAT, CCND1, CD36, CES3, CHKA, Cyp2c40 (includes others), Cyp2c54 (includes others), CYP2C8, DYNLL1, ELOVL5, FADS1, FOS, FTCD, G0S2, G6PD, GSTM3, GSTP1, H1-2, HPX, HSD3B1, IFITM3, LIPG, ME2, MGST1, MGST3, Mup1 (includes others), Orm1 (includes others), PLTP, PPARGC1A, QPCT, RTN4, SAA1, SCD, SELENBP1, TMEM98, UCP2

Table S2. Cisd2-mediated changes in the upstream transcription regulators and their downstream target genes in the liver of Cisd2TG mice.

Upstream Regulator	Molecule Type	Predicted Activation State	Activation z-score	p-value of overlap	Target molecules in dataset
TRIM24	transcription regulator	Activated	4.202	1.71E−17	CMPK2,DDX60,DHX58,Ifi47,IFIH1,IFIT1B,IFIT3,IRF7,LGALS3BP,Ly6a (includes others),NMI,PHF11,PLAC8,SAMD9L,SHISA5,STAT1,TAP1,USP18
**HNF4A**	transcription regulator	Activated	3.536	0.00239	ABCD2,ACOT1,CCND1,CYP3A5,FMO1,Hamp/Hamp2,HLA-A,MSN,NTRK2,RAP2B,RSAD2,Slco1a1,TRIM24
GSR	enzyme	Activated	2.449	0.000286	CBR1,CD36,GSTM1,HMOX1,MGST3,NQO1
TXNRD1	enzyme	Activated	2.425	0.000286	CBR1,CD36,GSTM1,HMOX1,MGST3,NQO1
SLC13A1	transporter	Activated	2.236	0.0397	ACTG1,APOA4,C9,INMT,Nat8f5
CD44	other	Inhibited	-2	0.0108	CCL5,CCR5,CD36,Ly6a (includes others)
**IL-6**	cytokine	Inhibited	−2.214	5.19E−07	ADGRE1,APCS,CCND1,CYP2C8,CYP3A5,Hamp/Hamp2,Orm1 (includes others),SAA1,Slco1a1
TNK1	kinase	Inhibited	−2.433	7.18E−07	IFI16,IFIH1,IFIT1B,IRF7,OAS2,TLR8
PPARA	ligand-dependent nuclear receptor	Inhibited	−2.523	3.17E−06	ABCD2,APCS,APOA4,C1QA,C1QB,C8A,C9,CCND1,CD36,CES3,Cyp2c54 (includes others),CYP2C8,G6PD,H1-2,HSD3B1,ME2,MGST3,Mup1 (includes others),Orm1 (includes others),RTN4,SAA1,SELENBP1,UCP2
**NFE2L2 (Nrf2)**	transcription regulator	Inhibited	−2.666	6.79E−05	ACTG1,ALAS2,APCS,APOA4,CBR1,CYP4A22,FMO1,GSTM1,HMOX1,HSD3B1,INMT,LY6E,MGST3,NQO1,SAA1,SERPINA3,UGT2B28
